# Volatile Oil from Amomi Fructus Attenuates 5-Fluorouracil-Induced Intestinal Mucositis

**DOI:** 10.3389/fphar.2017.00786

**Published:** 2017-11-09

**Authors:** Ting Zhang, Shan H. Lu, Qian Bi, Li Liang, Yan F. Wang, Xing X. Yang, Wen Gu, Jie Yu

**Affiliations:** College of Pharmaceutical Science, Yunnan University of Traditional Chinese Medicine, Kunming, China

**Keywords:** intestinal mucositis, Amomi Fructus, volatile oil, bornyl acetate, 5-fluorouracil

## Abstract

Amomi Fructus has been used to treat digestive diseases in the context of traditional Chinese medicine, so we evaluated the effects of a volatile oil from *Amomum villosum* (VOA) on intestinal mucositis induced by 5-fluorouracil (5-FU). We measured the effect of VOA and its main active constituent, bornyl acetate (BA), on body weight, food intake, diarrhea, inflammatory cytokines, the mucosal barrier, and gut microbiota. VOA and BA significantly increased the rats’ body weight, relieved diarrhea, and reversed histopathological changes in the gut and inflammation. VOA significantly inhibited apoptosis and alleviated the endoenteritis by downregulating p38 MAPK and caspase-3 expression. VOA and BA strengthened the intestinal mucosal barrier by increasing zonula occludin-1 and occludin expression. VOA and BA reduced the amount of pathogenic bacteria and increased the abundance of probiotics. Thus, VOA prevented the development and progression of intestinal mucositis after chemotherapy.

## Introduction

Intestinal mucositis, which is characterized by a decrease in villi length and the disruption of crypt cell homeostasis, is a common toxic side effect of the cancer chemotherapeutics 5-fluorouracil (5-FU) and irinotecan. This toxicity causes severe diarrhea and morphological mucosal damage, which limits the safety and clinical application of the drugs. Intestinal mucositis has many facets, including microstructural damage of small intestinal tissue, an injured intestinal mucosal barrier and inflammation. Small intestinal mucosal barrier integrity is frequently disrupted in various acute and chronic intestinal diseases (Turner, 2009), and tight junction proteins of the small intestinal mucosa, such as zonula occludin (ZO) and occludin, are needed to maintain the intestinal epithelial barrier (Suzuki, 2013). In addition, caspase and cysteine protease are needed for the morphological and biochemical changes that occur during apoptosis. Caspase-3, which is required for the activation of various apoptotic-stimulating factors, can target substrates and cause cell disassembly and DNA fragmentation (Flanagan et al., 2016). Damage to the small intestinal mucosal barrier can cause endotoxin-translocation-induced endotoxemia, which exacerbates inflammatory infiltration. Meanwhile, commensal intestinal microbiota may influence all of the phases of mucosal pathogenesis (van Vliet et al., 2010). Thus, treatment with prebiotics and probiotics may alleviate intestinal mucositis (Wada et al., 2010; Justino et al., 2014, 2015; Araújo et al., 2015). Efforts to reduce chemotherapy-induced intestinal damage, with traditional or conventional medicine have not been successful (Sonis, 2004; Cathy, 2010; Lam et al., 2010; Wang et al., 2014, 2015).

Amomi Fructus, the dry and mature fruit of *Amomum villosum* Lour., *A. villosum* Lour. var. *xanthioides* T. L. Wu et Senjen, and *A. longiligulare* T. L. Wu, has been recorded and used in traditional Chinese medicine as an excellent crude drug for the treatment of digestive system disorders (Commission of Chinese Materia Medica, 1999; Commission of Chinese Pharmacopoeia, 2015). Amomi Fructus is also authorized as a food by the China Food and Drug Administration^1^. Amomi Fructus has been shown to have a significant effect on the recovery of mice intestinal flora balance that was disturbed by antibiotics (Yan et al., 2013). In addition, aqueous extracts may improve and promote intestinal function (Huang and Zhou, 2006). However, whether Amomi Fructus works and its mechanism in the treatment of intestinal mucositis have yet to be elucidated. Therefore, we focused on the potential roles of volatile oil from *A. villosum* (VOA) and bornyl acetate (BA), the main component of VOA, on the regulation of intestinal microflora, the adjustment of the inflammatory process and oxidative stress, maintaining intestinal permeability, and alleviating the intestinal mucositis process to provide a more theoretical and scientific foundation for its clinic use.

## Materials and Methods

### Medicinal Materials

**FIGURE 1:**
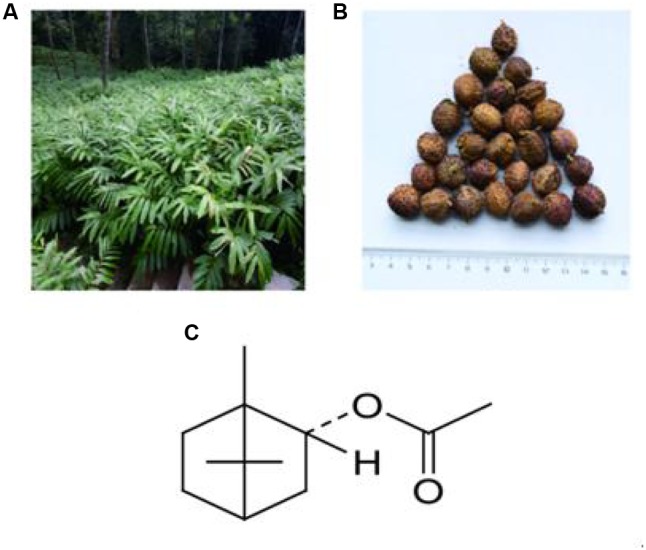
Plants used: **(A)** *Amomum villosum* Lour.; **(B)** fruit of *A. villosum* Lour.; and **(C)** structure of BA, the main constituent of VOA.

*Amomum villosum* Lour. (**Figures 1A,B**) was collected in Jingping County, Honghe Prefecture of Yunnan Province, China. The plants were identified as *A. villosum* Lour. by Jie Yu, an Associate Professor at Yunnan University of Traditional Chinese Medicine. Voucher specimens were deposited in the Herbarium of Pharmacognosy, Yunnan University of Traditional Chinese Medicine. BA (**Figure 1C**), was purchased from Jingzhu Biotechnology Co., Ltd. (Nanjing, China, purity > 98%, GC). 5-FU was obtained from XuDong HaiPu Pharmaceutical Co., Ltd. (Shanghai, China). Live combined *Bifidobacterium* and *Lactobacillus* tablets (CBL) were purchased from Inner Mongolia Shuangqi Pharmaceutical Co., Ltd. (China).

### Volatile Oil from *A. villosum*

Powder (100 g) of *A. villosum* fruit was soaked with 800 mL distilled water for 12 h (4°C). VOA, gathered by steam distillation, was stored at 4°C after drying with Na_2_SO_4_.

### GC-MS Characterization of VOA

Gas chromatography-mass spectrometry (GC-MS) was used to characterize VOA (HP6890GC/5973MS, Agilent). Chromatographic separation was achieved on an HP-5MS (30 mm × 0.25 mm, 0.25 μm) fused-silica capillary column. The injector temperature was 250°C and the column pressure was 100 kPa. Helium was used as the carrier gas (1.0 mL/min) and the injection volume was 0.1 μL (split ratio of 50:1). The temperature was 80°C for 2 min and increased to 280°C for 20 min (3°C/min).

Mass spectrometry conditions were as follows: ion source temperature, 230°C; electron energy, 70 eV; interface temperature, 250°C; quadrupole temperature, 150°C; and mass scan range, 30–500 amu. Wiley7n.l mass spectral database was used to compare data with a standard spectrum to identify the components of every peak.

### Animals

Fifty-four, 8-week-old male Spraque–Dawley rats (Da Shuo Biotech Co., Ltd., Chengdu, China, Certificate of Quality No: 0016254) were kept in a temperature-controlled room with free access to water and food, and they fasted for 2 h before all of the treatments. The study was approved by the Institutional Ethical Committee on Animal Care and Experimentation of Yunnan University of Traditional Chinese Medicine (R-0620150028). All reasonable efforts were made to minimize animal suffering.

### Rats Model of Intestinal Mucositis Induced by 5-FU

After adaptive feeding for 3 days, the rats were randomized to one of nine groups (*N* = 6/group): normal controls (CON); 5-FU-induced intestinal mucositis model (MOD); live combined *Bifidobacterium* and *Lactobacillus* tablets (CBL, 450 mg/kg) positive controls; VOA low, medium, and high groups (VOA.L, 8 mg/kg; VOA.M, 16 mg/kg; and VOA.H, 32 mg/kg); BA low, medium, and high groups (BA.L, 2 mg/kg; BA.M, 4 mg/kg; and BA.H, 8 mg/kg). All of the rats, except for the normal controls, received 5-FU (35 mg/kg, daily, i.p.) for 5 days. Researchers used personal protective gear to prevent exposure to 5-FU and CBL. At the end of the study, the drugs were destroyed by the Experimental Center of Yunnan University of Traditional Chinese Medicine.

**FIGURE 2:**
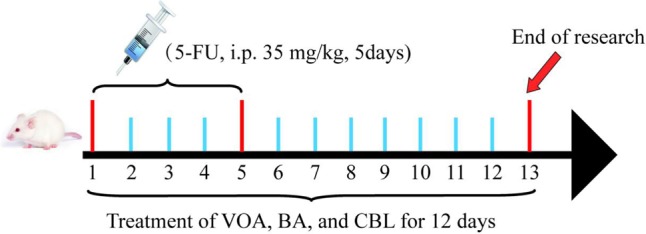
Treatment schedule. All of the rats, except for the normal controls, received 5-FU (35 mg/kg daily, i.p.) for 5 days. Then, 4 h before chemotherapy, rats received CBL, VOA, or BA treatments (via gavage) once daily for 12 days.

The dose of *A. villosum* was 3–6 g/kg in clinical medium and the BA concentration was required to be not less than 1% in *A. villosum* according to the Commission of Chinese Pharmacopoeia (2015). The yield of VOA was 3.9% from *A. villosum* in our study and BA was calculated as 1%, which was the minimum content of BA regulated by the Commission of Chinese Pharmacopoeia (2015). The clinical dose for humans was calculated as 4.5 g/kg in this study. These factors were considered in our dosage conversation, and we then converted the human dosage into the rat dose according to the body surface area. We concluded that the dose of VOA was 16 mg/kg and BA was 4 mg/kg in rats, which was considered to be the medium dose. Finally, the low, medium, and high dose of each administration group was calculated by 1:2:4. VOA and BA were dissolved in distilled water with 0.1% Tween-80 by an ultrasonic mixer at 16°C and then stored at 4°C. Before chemotherapy (4 h), the rats received CBL, VOA, and BA (via gavage) once daily for 12 days. **Figure 2** depicts the treatment schedule.

Whether or not the rats had diarrhea, and their weight and food intake were recorded daily. Rats were killed 12 days after treatment using 10% chloral hydrate anesthetization. Blood samples and small intestinal tissue were collected.

### Morphology and Histopathology Observation of Small Intestinal Tissue

Segments of the jejunum were collected, fixed, and stained with hematoxylin and eosin to measure the villus height and crypt depth. Three tissues from different rats in each group and three images (10×) per section were analyzed for morphological studies.

### IL-6, ROS, TNF-α, NF-κB, and MPO in Blood Samples and Small Intestinal Tissue

Blood samples were collected from the retro-orbital venous plexus and hepatic portal vein at 2 h after treatment in the morning at the end of the study. Serum was centrifuged at 12,000 × *g* for 15 min and analyzed immediately. Serum interleukin-6 (IL-6) and reactive oxygen species (ROS) were measured with ELISA kits purchased from Cusabio Biotech Co., Ltd. (China).

At the end of the treatment, the rats were killed via 10% chloral hydrate (0.3 mL/100 g, ip). Small intestine tissue samples were excised, weighed, and washed in 0.9% saline. Tissues (100 mg) were rinsed with PBS and homogenized in 1 mL of PBS and then stored overnight at -20°C. Two freeze-thaw cycles were performed to break the cell membranes, and homogenates were centrifuged for 10 min at 6,500 × *g*, 4°C. The supernatant was collected for biochemical analysis. ROS, tumor necrosis factor-α (TNF-α), nuclear factor of kappa b (NF-κB), and myeloperoxidase MPO in the intestinal homogenate were measured with ELISA kits purchased from Cusabio Biotech Co., Ltd. (China).

### Hepatic Portal Vein Lipopolysaccharide (LPS)

Hepatic portal vein blood samples were centrifuged at 6,500 × *g* for 10 min at 4°C after being collected under anesthesia. Lipopolysaccharide (LPS) was measured using a tachypleus amebocyte lysate test purchased from Chinese Horseshoe Crab Reagent Manufacturers Co., Ltd. (Xiamen, China).

### Flow Cytometric Measurement of Occludin, ZO-1, and Caspase-3

A single-cell suspension was prepared by cutting and disrupting one representative intestinal sample from each group through a 70-μm filter membrane. Cells were diluted with staining buffer (1 × 10^6^ cells/mL) after blockage with 3% FBS. For analysis, the cells were incubated with an anti-occludin antibody (EPR8208, Abcam, United States), anti-ZO-1 antibody (21773-1-AP, Proteintech, United States) and an anti-caspase antibody (25546-1-AP, Proteintech, United States) for 2 h, and then they were incubated with fluorescein isothiocyanate (FITC, SA00003-2) in the dark for 1 h. Occludin, ZO-1, and caspase-3 expression was measured using flow cytometry (FACSCalibur, Becton, Dickinson, and Company, United States).

### Western Blot for MAPK

Intestinal tissues (100 mg) were homogenized using 1 mL PIRA lysis buffer, and centrifuged (12,000 × *g*, 10 min, 4°C). Then, samples were kept on ice for 20 min. The supernatant was collected for Western blotting, and the extracted proteins were quantified using the bicinchoninic acid (BCA) method. Next, 50 μg of total protein was added to each sample. Proteins were resolved with 10% sodium dodecyl sulfate (SDS)-polyacrylamide gels (PAGE) and transferred to polyvinylidene fluoride (PVDF) membranes. Transferred membranes were blocked with 5% albumin bovine V (BSA) to inhibit non-specific proteins. Then, the primary antibodies (9212S, 1:1.000 dilution; Cell Signaling, Danver, MA, United States) were added, with β-actin (20536-1-AP, 1:1.000 dilution; Proteintech Group, United States) used as an internal reference. After incubating with primary antibodies, the membranes were washed with TBS/Tween-20 (TBST) three times and then incubated with the secondary antibodies (SA00001-2, 1:10.000 dilution; Proteintech Group, United States). Each protein band was visualized with an enhanced chemiluminescence (ECL) detection system (Proteintech Group, United States) and quantified with Quantity One Analysis Software (Bio-Rad Laboratories, Inc.).

### 16S rDNA Gene Sequencing of Rat Feces

Rat feces samples were collected on the last day and placed in a sterile centrifuge tube. They were then evenly ground in liquid nitrogen and preserved at -80°C for future inspection. All of the fecal samples from the treatment groups were blended, and transferred to a mortar and ground to a fine powder. DNA was extracted according to the instructions of the stool DNA kit (Omegea Bio-tek, United States).

To determine bacterial diversity and composition in the feces samples, we used the protocol described by Caporaso’s group (Caporaso et al., 2010). PCR amplifications were conducted with a 515f/806r primer set that amplifies the V4 region of the 16S rDNA gene. This primer set has few biases and yields accurate phylogenetic and taxonomic information. The reverse primer contains a 6-bp error-correcting barcode unique to each sample. DNA was amplified as described in the literature (Magoč and Salzberg, 2011). Sequencing was conducted on an Illumina MiSeq platform (Novogene, Beijing, China).

### Statistical Analysis

Data are means ± SD. One-way analysis of variance (ANOVA) was used to compare multiple groups (*p* < 0.05, < 0.01, and < 0.001). Graphics were created with Origin 6.1 (MicroCal Software, United States). A 95% confidence interval (CI) was used as a threshold to identify potential outliers in all of the samples, and clustering was analyzed with TMEV Clustering (Mev Development Team).

## Results

### *A. villosum* Lour. Characterization

A total of 65 compounds were determined, and 58 compounds were successfully identified, which represented over 99% of the total oil composition. As shown in **Table 1**, BA (54.54%), camphor (17.92%), camphene (6.76%), limonene (5.25%), borneol (4.07%), myrcene (1.97%), α-pinene (1.50%), β-caryophyllene (0.85%), β-pinene (0.80%), and α-pepperene (0.54%) were detected in VOA. BA was considered as the representative compound of VOA.

**Table 1:** Constituents of VOA.

No.	Compounds	MW	Formula	*t*/min	Area (%)
1	Bornyl acetate	196	C_12_H_20_O_2_	13.266	54.537%
2	Camphor	152	C_10_H_16_O	8.415	17.921%
3	Camphene	136	C_10_H_16_	3.911	6.757%
4	Cinene	136	C_10_H_16_	5.295	5.249%
5	Borneol	154	C_10_H_18_O	8.986	4.068%
6	Myrcene	136	C_10_H_16_	4.462	1.969%
7	α-Pinene	136	C_10_H_16_	3.666	1.503%
8	β-Caryophyllene	204	C_15_H_24_	18.106	0.853%
9	β-Pinene	136	C_10_H_16_	4.344	0.795%
10	α-Copaene	204	C_15_H_24_	16.434	0.543%

### General Condition of Experimental Animals

**FIGURE 3:**
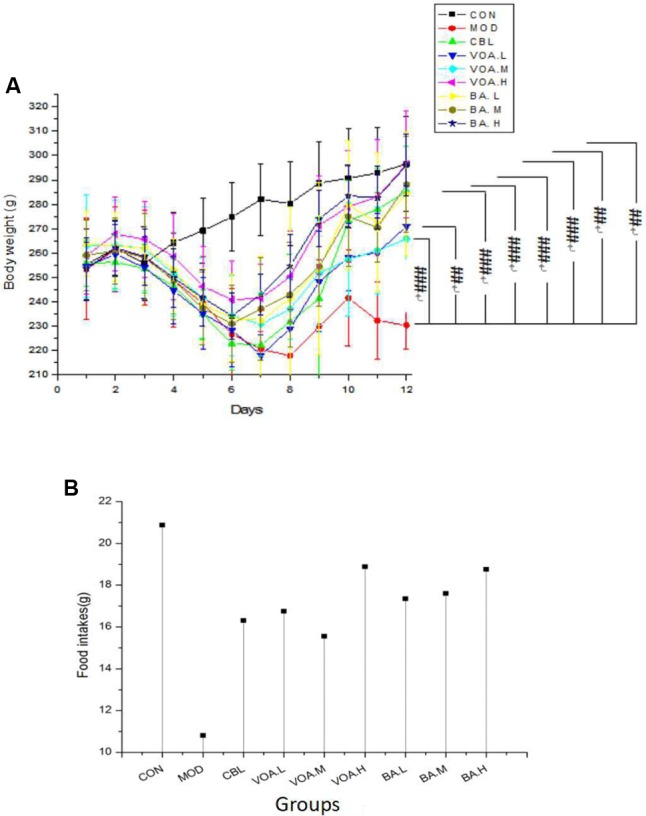
Weight changes **(A)** and mean food intake **(B)** across groups. Data were recorded daily, ^#^ significant difference compared to the model and controls at the end of the study. *^#^p* < 0.05, *^##^p* < 0.01, and *^###^p* < 0.001.

Initial animal weight and food intake were similar among the groups, and the controls gained weight over time (**Figure 3A**). Rats treated with 5-FU had decreased weight from the fourth to the eighth day and the weight of these animals was the lowest until the end of the experiment. Treatment with CBL, VOA, and BA prevented (*p* < 0.01) significant weight loss in the MOD group. During the study, the average MOD food intake was 10.81 g/day, which was roughly half of the normal group (**Figure 3B**). CBL, VOA, and BA treatments reversed the appetite reduction in the MOD group. Compared with the MOD group, the food intake of the CBL, VOA, and BA groups was increased by about 50%.

### VOA Improved Morphology and Microstructures of Small Intestinal Tissue

**FIGURE 4:**
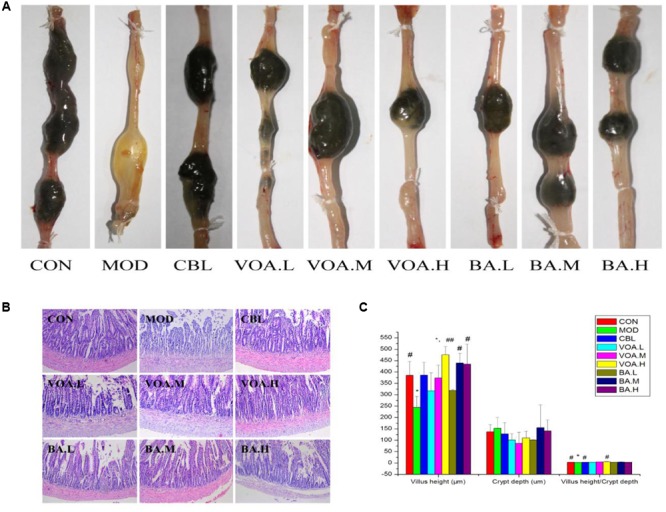
**(A)** VOA and BA reduced diarrhea after 5-FU induced intestinal mucositis. Stool from the 5-FU group and other treatment groups. **(B)** Reduced intestinal damage after VOA and BA treatment. Photomicrographs (10×) of the jejunums from the control, model, and treatment groups (*n* = 3). 5-FU shortened villi, deepened crypts, and increased inflammatory cell infiltration. VOA and BA reduced this side effect. **(C)** Reversion of 5-FU-induced intestinal morphometric changes by VOA and BA in rats, *n* = 3. Segments of jejunum retrieved to measure villus height, crypt depth and the villus:crypt ratios. ^∗^Significant difference compared to the controls. Values are means ± SD. ^#^ Significant difference compared to the model and controls. *^∗^p* < 0.05, *^#^p* < 0.05, and *^##^p* < 0.01.

Fecal appearance and shape indicated diarrhea in the MOD group, and all of the treatments relieved 5-FU-induced watery diarrhea (**Figure 4A**). Compared with the controls, the MOD group had histopathological changes in the jejunum, such as mucosa with shortened villi with vacuolated cells, crypt necrosis, and intense inflammatory cell infiltration. Treatment with VOA, BA, and CBL for 12 days reversed these changes in limited fashion (**Figure 4B**).

The administration of 5-FU induced significant decreases in villus height, increased crypt depth, and decreased villus: crypt ratio (**Figure 4C**). **Figure 4C** shows that CBL, VOA, and BA treatment reversed 5-FU-induced reductions in villus height. Similarly, a smaller villus: crypt ratio was observed in the MOD group, and this was reversed with the drug treatments, especially with the VOA.H treatment (*p* < 0.01).

### VOA Prevented Serum and Intestinal Inflammation

**FIGURE 5:**
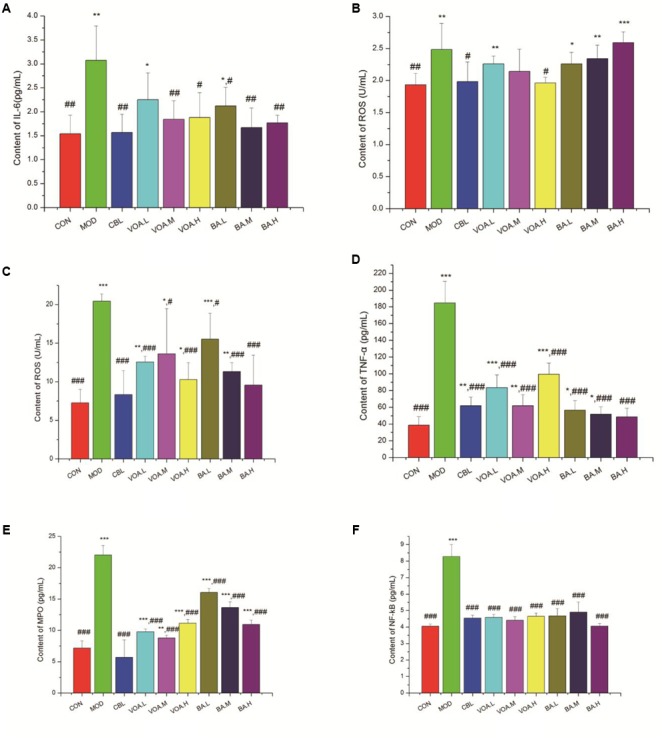
Reduced IL-6, ROS, TNF-α, NF-κB, and MPO after VOA and BA treatment in blood and small intestinal tissue (*n* = 6). Increased **(A)** IL-6 and **(B)** ROS in blood after 5-FU. VOA and BA reduced inflammation. Increased **(C)** ROS, **(D)** TNF-α, **(E)** MPO, and **(F)** NF-κB in small intestinal tissue after 5-FU. VOA and BA reduced pro-inflammatory cytokines. Values are means, with their standard deviation represented by vertical bars. Values are means ± SD. ^∗^ Significant difference compared to the controls. ^#^ Significant difference compared to the model and controls. *^∗^p* < 0.05, *^∗∗^p* < 0.01, *^∗∗∗^p* < 0.001, *^#^p* < 0.05, *^##^p* < 0.01, and *^###^p* < 0.001.

After an injection of 5-FU, we observed a significant increase (*p* < 0.01) in the serum IL-6 concentration (3.07 ± 0.79 pg/mL) compared with the controls (1.54 ± 0.39 pg/mL), suggesting that the inflammation was elevated in the MOD rats. Treatment with VOA and BA significantly reduced the IL-6 levels by 26.71%, 40.06%, 38.76%, 30.94%, 45.60%, and 42.34% (**Figure 5A**). In the meantime, we found a significantly elevated ROS concentration in the sera of the MOD group (2.48 ± 0.41 U/mL) compared with the control group (1.93 ± 0.18 U/mL); this indicated that the model rats induced by 5-FU were in an inflammatory status. The increase was significantly inhibited after treatment with CBL (1.98 ± 0.31 U/mL) (**Figure 5B**). In brief, VOA.M, VOA.H, and BA suppressed the 5-FU-mediated increase in IL-6, and VOA.H suppressed the ROS increase compared with the MOD group.

As shown in **Figures 5C,D**, the concentrations of ROS and TNF-α in the small intestinal tissue of the intestinal mucositis rats was increased significantly (to 20.42 ± 1.10 U/mL and 184.7442 ± 25.71 pg/mL, respectively). Similarly, MPO and NF-κB concentrations were also increased by 67.44% and 51.21%, respectively, in the intestinal mucositis rats. All of the treatments could effectively reduce (*p* < 0.001) these inflammatory factor levels. **Figure 5C** shows that treatment with BA significantly reduced ROS concentrations by 24.06%, 44.62%, and 53.18%, while **Figure 5D** shows that treatment with BA significantly reduced TNF-α concentrations by 69.43%, 72.02%, and 73.77%, respectively. VOA treatment reduced MPO (55.63%, 60.03%, and 49.46%, respectively) and NF-κB concentrations (44.68%, 46.74%, and 43.84%, respectively) (**Figures 5E,F**). Thus, VOA and BA prevented inflammation in the small intestinal tissue after 5-FU chemotherapy.

### VOA Downregulated p38 MAPK and Caspase-3 Protein Expression

**FIGURE 6:**
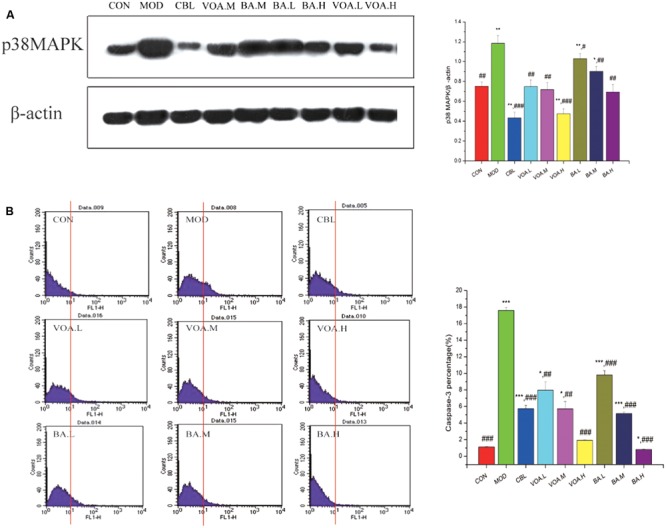
**(A)** Reduction of p38 MAPK expression by VOA and BA in small intestinal tissue after 5-FU treatment, *n* = 6. 5-FU increased the p38 MAPK expression compared to the controls. GLB, VOA, and BA reduced p38 MAPK (*p* < 0.001, *p* < 0.01, and *p* < 0.001). **(B)** Reduction of elevated caspase-3 by VOA and BA in small intestinal tissue after 5-FU treatment, *n* = 6. In the histogram, the data of the horizontal coordinates that were more than 10^1^ were counted and were used as original data to draw the bar chart. Histogram of data from 5-FU shows significantly increased caspase-3 expression compared to the controls. VOA or BA reduced caspase-3 (*p* < 0.01 and *p* < 0.001). Values are means ± SD. ^∗^ Significant difference compared to the controls. ^#^ Significant difference compared to the model and controls. ^∗^*p* < 0.05, ^∗∗^*p* < 0.01, *^∗∗∗^p* < 0.001, *^#^p* < 0.05, *^##^p* < 0.01, and *^###^p* < 0.001.

The concentrations of p38 MAPK in the small intestine were detected by Western blotting. Our results revealed that p38 MAPK expression in the MOD group was significantly higher than the CON (control) group (*p* < 0.01), while the therapeutic drugs could successfully inhibit the expression of p38 MAPK. Treatment with VOA and BA reduced (*p* < 0.01) the p38 MAPK levels by 37.29%, 39.83%, 60.17%, 12.71%, 23.73%, and 41.53%, respectively (**Figure 6A**).

Caspase-3 protein in the small intestinal tissue was measured with flow cytometry. **Figure 6B** shows that 5-FU significantly increased the expression of caspase-3 compared with the control. In contrast, both VOA and BA could effectively reduce (*p* < 0.01 and *p* < 0.001) the expression of caspase-3 in a dose-dependent manner. These data suggested that they could significantly inhibit cell apoptosis and alleviate the development of endoenteritis.

### VOA Strengthened the Intestinal Mucosal Barrier and Inhibited Enterogenous Endotoxin

**FIGURE 7:**
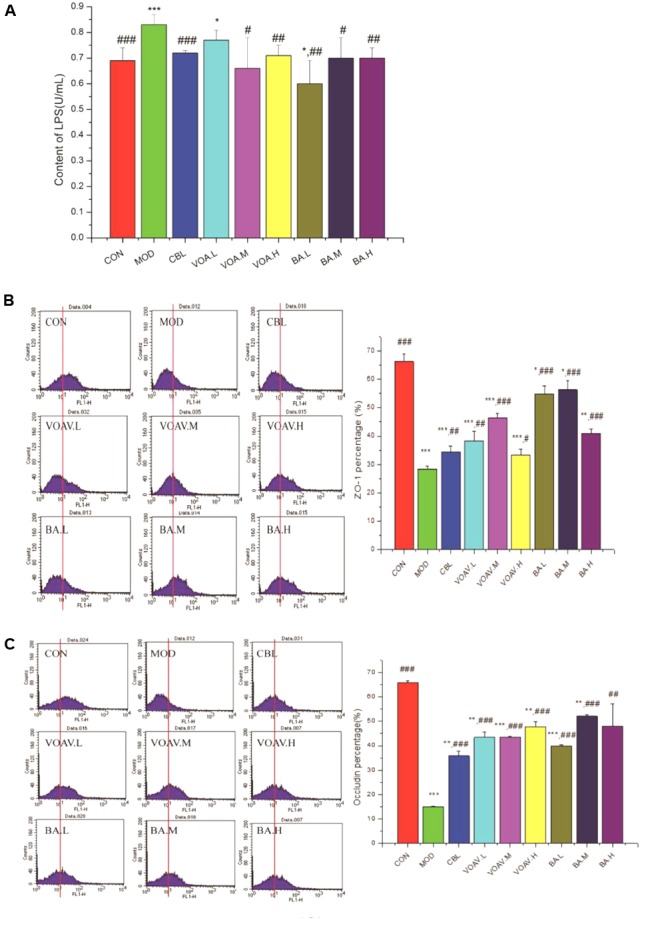
Effects of VOA on enterogenous endotoxin and intestinal mucosal barrier regulation. **(A)** LPS in hepatic portal veins, *n* = 6 was increased after 5-FU. VOA and BA blocked increases in LPS, **(B)** ZO-1, *n* = 6, and **(C)** occludin, *n* = 6. In the histogram, the data of the horizontal coordinate that were more than 10^1^ were counted and were used as original data to draw the bar chart. The histograms of ZO-1 and occludin show a decrease after 5-FU. VOA and BA increased proteins (*p* < 0.01 and *p* < 0.001). Values are means ± SD. ^∗^ Significant difference compared to the controls. ^#^ Significant difference compared to the model and controls. *^∗^p* < 0.05, *^∗∗^p* < 0.01, *^∗∗∗^p* < 0.001, *^#^p* < 0.05, *^##^p* < 0.01, and *^###^p* < 0.001.

The 5-FU treatment increased LPS in the MOD group more than CON group (*p* < 0.001; **Figure 7A**). VOA (*p* < 0.01) and BA (*p* < 0.05) showed a satisfactory effect with respect to the inhibition of LPS rise. This inhibition effect may be related to its beneficial effects on gut microbiota equilibrium.

**Figures 7B,C** show that the expression of ZO-1 and occludin was significantly lower (*p* < 0.001) in MOD rats compared to the CON rats, and this agreed with a previous study (Song et al., 2013). VOA and BA increased ZO-1 and occludin protein expression to normal levels. VOA and BA improved the intestinal mucosal barrier by increasing the expression of ZO-1 and occludin protein and blocking endotoxin translocation-induced endotoxemia.

### VOA Regulated Intestinal Microbial Balance and Altered Its Structure and Composition

**FIGURE 8:**
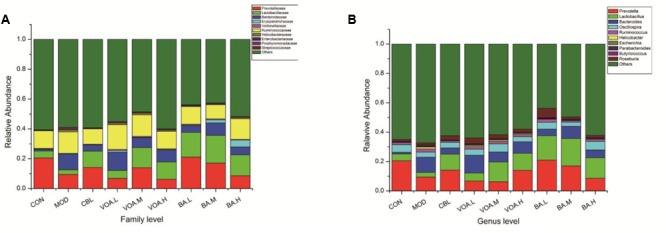
Relative abundance of gut microbiota with respect to family **(A)** and genus **(B)** after 5-FU-induced intestinal mucositis.

The structure and composition of gut microbiota were dramatically altered by 5-FU injection. Results of the microbial classification at the level of family showed that the relative abundance of Bacteroidaceae, Helicobacteraceae, Enterobacteriaceae, Porphyromonadaceae, and Streptococcaceae in the CON group was 1.19%, 0.32%, 0.02%, 0.25%, and 0.02%, respectively. However, their relative abundance in the MOD group increased to 10.40%, 1.54%, 0.28%, 1.09%, and 0.07%, respectively. The relative abundance of Helicobacteraceae, Enterobacteriaceae, Porphyromonadaceae, and Streptococcaceae was significantly reduced after treatment with BA, and Bacteroidaceae was reduced by VOA.M, VOA.H, and BA. Meanwhile, the relative abundance of Lactobacillaceae, which are usually considered probiotics, decreased from 4.69% in the CON group to 3.15% in the MOD group. VOA and BA treatment reincreased the abundance of Lactobacillaceae to 5.35–13.45% and 14.03–18.63%, respectively (**Figure 8A**).

Results of the microbial classification at the level of genus showed that the relative abundance of the *Bacteroides* (Bacteroidaceae), *Ruminococcus* (Ruminococcaceae), *Helicobacter* (Helicobacteraceae), *Escherichia* (Enterobacteriaceae), and *Parabacteroides* (Porphyromonadaceae) in the CON group was 1.19%, 1.48%, 0.03%, 0.01%, and 0.25%, respectively. However, their relative abundance in the MOD group increased to 10.40%, 2.14%, 1.24%, 0.28%, and 1.09%, respectively. The relative abundance of *Ruminococcus, Helicobacter, Escherichia*, and *Parabacteroides* was significantly reduced after treatment with BA, and *Bacteroides* was reduced by VOA.M, VOA.H, and BA. The relative abundance of *Lactobacillus* (Lactobacillaceae) in the CON group was 4.69%, and it decreased to 3.15% in the MOD group. After treatments with VOA and BA, the abundance of *Lactobacillus* increased to 5.35–13.45% and 14.03–18.63%, respectively (**Figure 8B**).

**FIGURE 9:**
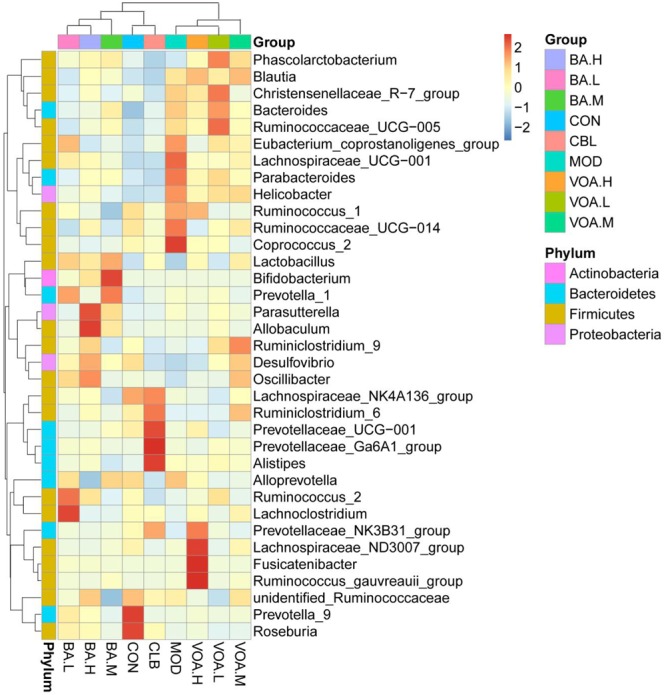
Species abundance clustering at the genus level. The *X*-axis represents sample information, the *Y*-axis represents annotation information for species, the clustering tree on the left is the species clustering, and the upper area is the sample clustering. Center values are *Z*-values standardized for species abundance in each line.

In addition, according to the species annotations and the abundance information at the level of genus, the heat map of the most abundant 35 genus was plotted and cluster analysis was conducted (**Figure 9**). They could be divided into four categories: BA.L/BA.M/BA.H; CON/CBL; MOD/VOA.H/VOA.L; and VOA.M/BA, and we found that BA.M and CBL were the closest. We concluded that BA increased the abundance of probiotics, such as *Lactobacillus* and *Bifidobacterium*, while VOA increased the abundance of *Roseburia* and *Coprococcus*. We suggested that BA and VOA might regulate intestinal microecology in two ways not just one way.

**FIGURE 10:**
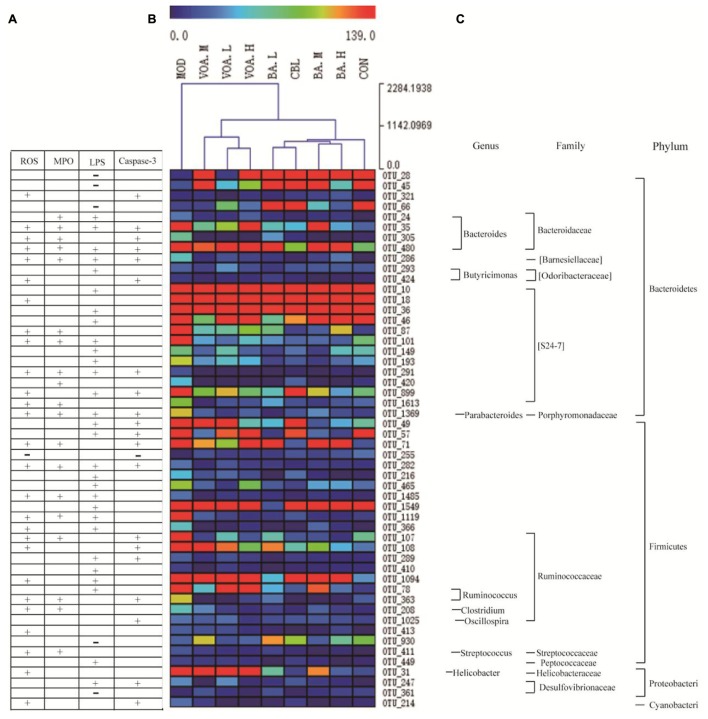
Correlation between 52 key OTUs and intestinal mucositis index. These key OTUs were significantly altered after VOA and BA treatments, and were correlated with changes in ROS, MPO, LPS, and caspase-3 **(A)**. Clustering analysis of OTU **(B)** and represented bacteria taxa information (genus, family, and phylum) of 52 key OTUs **(C)**. “+” Indicates a positive correlation with ROS, MPO, LPS, and caspase-3, and “–” indicates a negative correlation. Attachment

Pearson correlation and cluster analyses were performed with clean operational taxonomic unit (OTU) data and we identified 52 key variables, which were significantly altered after the VOA and BA treatments. These were correlated to alternations in ROS, MPO, LPS, and caspase-3 (**Figure 10**). Among them, Bacteroidaceae (four OTUs, from Bacteroidetes) were positively correlated with ROS, MPO, and caspase-3. S24-7 (eight OTUs, from Bacteroidetes), Ruminococcaceae (five OTUs, from Firmicutes), and Desulfovibrionaceae (one OTU, from Proteobacteria) were positively correlated with LPS. In addition, a few bacteria from Bacteroidetes (three OTUs) and Firmicutes (one OTU) were negatively correlated with LPS (**Figures 10A,C**).

Clustering analysis of the OTUs (**Figure 10B**) showed that among the 52 key OTUs, most of them in the MOD rats were increased when compared to the CON group. Using the Euclidean distance metric, the MOD group was at a long distance from the CON group. However, all of the treatment groups could shorten the distance to the CON group and the VOA and BA groups were clustered into two trees, suggesting that VOA and BA had a significant effect on the regulation of the 52 key OTUs. Thus, gut microbiota in the MOD rats were significantly altered compared to CON rats, and CLB, VOA, and BA treatment could partially recover the gut microbiota equilibrium.

## Discussion and Conclusion

The VOA and BA treatments prevented the occurrence of diarrhea and reversed weight loss and diminished food intake, all symptoms of 5-FU-induced intestinal mucositis. In addition, VOA and BA treatments significantly improved the histopathological changes in rat intestinal mucositis induced by 5-FU, perhaps due to a reduction in inflammatory ROS, IL-6, TNF-α, and NF-κB, decreased MPO, decreased p38 MAPK and caspase-3 proteins, and improved intestinal mucosal barrier function. VOA and BA also contributed to the regulation of the intestinal microbiota balance.

Intestinal injury may induce p53/PUMA-mediated apoptotic, brush-border hydrolase activity changes, blunted villus height, crypt deepening, and increased crypt cell apoptosis with decreased proliferation (Zhan et al., 2014). Chemotherapy drugs often indiscriminately damage DNA, tumor cells, and normal cells, causing increased ROS and reactive nitrogen species (RNS). They may also activate downstream signaling pathways to induce local inflammatory responses and cause damage to intestinal epithelial cells (Sonis, 2004). ROS, which are crucial mediators of downstream biological events, are then produced. VOA appeared to block 5-FU-induced increases in ROS in blood and the small intestines, and these data agreed with a report by Arifa’s group (Arifa et al., 2014), who observed that inflammasome activation was dependent on ROS and that anti-neoplastic drugs induced mucositis via inflammatory factors in mice.

DNA damage, non-DNA damage, and ROS activate several transduction pathways that then activate transcription factors, such as NF-κB (Sonis, 2002). NF-κB activates downstream genes, including LPS and pro-inflammatory cytokines, such as TNF-α, IL-6, and IL-1 (Rinkenbaugh and Baldwin, 2016). In the present study, VOA and BA significantly reduced the expression of NF-κB and reduced pro-inflammatory cytokines (IL-6 and TNF-α) that had been increased by 5-FU; this suggested that VOA and BA play protective roles in the inflammatory state.

p38 MAPK, an important member of the MAPK family, participates in cell proliferation, apoptosis, and differentiation (Horie et al., 2012; Chen et al., 2014; Lee et al., 2014). VOA and BA treatment inhibited MAPK and capase-3 signaling and suppressed MPO activity, decreased apoptosis, and protected the integrity of the intestinal mucosal barrier. BA had a dose-dependent relationship with the regulation of ROS, TNF-α, MPO, and related proteins (p38 MAPK and caspase-3). VOA had a dose-dependent relationship with respect to the regulation of p38 MAPK and caspase-3. This may be due to different the compositions of VOA and BA.

Lipopolysaccharide, a cell component of Gram-negative bacteria, is delivered to the liver via the portal vein in endotoxemia (Lin et al., 2015). Endotoxin leads to chronic low-grade inflammation in patients with intestinal mucositis. LPS is thought to disrupt the epithelial barrier integrity by downregulating the tight junction proteins (Sheth et al., 2007; Han et al., 2016). We measured the tight junction, ZO-1, and occludin proteins in the small intestinal tissue and LPS in the hepatic portal veins. There is substantial evidence suggesting that tight junctions play pivotal roles in the pathophysiology of chemotherapy drug-induced gut toxicity, so we hypothesized that VOA could improve 5-FU-induced small intestinal mucositis, which may be related to changes in tight junction protein expression and reduced LPS, and it is perhaps mediated by the MAPK and NF-κB pathways.

We also measured the influence of commensal intestinal microbiota, such as *Escherichia, Bacteroides, Helicobacter, Desulfovibrio, Ruminococcus, Parabacteroides*, and *Clostridium*. Cassmann’s group (Cassmann et al., 2016) found that dogs with chronic enteropathies harbored more mucosal bacteria from *Bacteroides, Escherichia*, and Enterobacteriaceae. Studies show that *Helicobacter pylori* is an important factor in gastroduodenal diseases as *H. pylori* infection leads to chronic gastritis in children and adults (Mansour-Ghanaei et al., 2010). Asonum’s group reported that *H. pylori* is a major cause of transdifferentiation into intestinal metaplasia that causes gastric cancer (Asonuma et al., 2009). *Desulfovibrio* was significantly increased in acute and chronic ulcerative colitis at multiple levels within the colon (Rowan et al., 2010). *Ruminococcus* is a symbiotic anaerobic bacteria present in the gastrointestinal tract and its overgrowth occurs in inflammatory bowel disease (IBD). In patients with irritable bowel syndrome, there were fewer *Bifidobacterium* and more *Ruminococcus* and *Bacteroides* abundances than healthy (Shukla et al., 2015). Dziarski’s group (Dziarski et al., 2016) found that *Parabacteroides* and *Bacteroides* promoted colitis. *Clostridium* perfringens is a Gram-positive anaerobic pathogen that is usually associated with skin and soft tissue infections, gastrointestinal infections, and occasionally bacteremia (Antony et al., 2009).

In our study, BA and VOA significantly reduced *Escherichia, Bacteroides, Helicobacter, Desulfovibrio, Ruminococcus, Parabacteroides*, and *Clostridium*, which are pathogenic bacteria. BOA increased *Lactobacillus* and *Bifidobacterium*, the latter of which has been shown to decrease NF-κB activation (Beg, 2004), leading to decreased endotoxin and plasma IL-6 (O’Hara et al., 2006). *Lactobacillus* facilitates the maintenance of the intestinal membrane integrity during *Shigella dysenteriae* 1 infection in rats (Moorthy et al., 2009). VOA increased *Roseburia* and *Coprococcus* compared with the MOD rats, and study suggest that *Roseburia, Coprococcus*, and *Ruminococcus* were significantly reduced, whereas pathogens *Escherichia* and *Enterococcus* were prevalent in patients with IBD (Chen et al., 2014). This study support our findings.

Furthermore, we identified 52 key variables that were significantly altered after VOA and BA treatments, and clustering analysis suggested that the VOA and BA treatments partially recovered gut microbiota equilibria. In addition, BA or VOA dose effects were clustered together, while BA or VOA were in a wide range; this might be due to the fact that VOA is a mixture and BA is a monomeric compound. To the best of our knowledge, this is the first study that evaluates the effects of VOA on the intestinal epithelial barrier, inflammation, and the intestinal microbial imbalance induced by 5-FU. We hope these data will inform future novel probiotic-based treatments for intestinal toxicity associated with anticancer therapy. It is the theoretically possible that VOA or BA could interfere with the anticancer effect of 5-FU, and thus reduce the side effects of 5-FU. However, other study indicated that 5-FU retained its anti-cancer efficacy when used in combination with other medicinal products in different cancer models. The combined effects of sinomenine and 5-FU on esophageal carcinoma were superior to those of the individual compounds, and the drug combination did not increase the side effects of chemotherapy (Wang et al., 2013). Definitely, the benefit-risk profile of the combined treatment of 5-FU with VOA/BA is also worth studying. We would like to find these answers in our future research.

## Author Contributions

TZ and SL conducted the study, analyzed the data, and prepared the manuscript. QB, LL, and YW collected and interpreted the data. XY, WG, and JY designed the study, provided the drugs and research facilities, and revised the manuscript.

## Conflict of Interest Statement

The authors declare that the research was conducted in the absence of any commercial or financial relationships that could be construed as a potential conflict of interest.

## Footnotes

1 http://www.nhfpc.gov.cn/ewebeditor/uploadfile/2013/07/20130712155225821.doc

## References

[B1] Antony N. Westbrook R. Antony S. (2009). Clostrium perfringes infection of a total knee arthroplasty case report and review of the literature. *Internet J. Infect. Dis.* 8 1–3. 10.1016/j.semarthrit.2015.09.009 26546506

[B2] Araújo C. V. Lazzarotto C. R. Aquino C. C. Fiqueiredo I. L. Costa T. B. Alves L. A. (2015). Alanyl-glutamine attenuates 5-fluorouracil-induced intestinal mucositis in apolipoprotein E-deficient mice. *Braz. J. Med. Biol. Res.* 48 493–501. 10.1590/1414-431X20144360 25945744 PMC4470307

[B3] Arifa R. D. Madeira M. F. de Paula T. P. Lima L. Tavares L. D. Garcia Z. M. (2014). Inflammasome activation is reactive oxygen species dependent and mediates irinotecan-induced mucositis through IL-1βand IL-18 in mice. *Am. J. Pathol.* 184 2023–2034. 10.1016/j.ajpath.2014.03.012 24952429

[B4] Asonuma S. Imatani A. Asano N. Oikawa T. Konishi H. Iijima K. (2009). *Helicobacter* pylori induces gastric mucosal intestinal metaplasia through the inhibition of interleukin-4-mediated HMG box protein Sox2 expression. *Am. J. Physiol. Gastrointest. Liver Physiol.* 297 312–322. 10.1152/ajpgi.00518.2007 19520737

[B5] Beg A. A. (2004). ComPPARtmentalizing NF-kappaB in the gut. *Nat. Immunol.* 5 14–16. 10.1038/ni0104-14 14699401

[B6] Caporaso J. G. Kuczynski J. Stombaugh J. Bittinger K. Bushman F. D. Costello E. K. (2010). QIIME allows analysis of high-throughput community sequencing data. *Nat. Methods* 7 335–336. 10.1038/nmeth.f.303 20383131 PMC3156573

[B7] Cassmann E. White R. Atherly T. Wang C. Sun Y. Khoda S. (2016). Alterations of the ileal and colonic mucosal microbiota in canine chronic enteropathies. *PLOS ONE* 11:e014732. 10.13371/journal.pone.0147321 26840462 PMC4740465

[B8] Cathy E. (2010). Are herbal medicines ripe for the cancer clinic? *Sci. Transl. Med.* 2 41–45. 10.1126/scitranslmed.3001517 20720215

[B9] Chen J. Y. Zhang L. Zhang H. Su L. Qin L. P. (2014). Triggering of p38 MAPK and JNK signaling is important for oleanolic acid-induced apoptosis via the mitochondrial death pathway in hypertrophic scar fibroblasts. *Phytother. Res.* 28 1468–1478. 10.1002/ptr.5150 24706573

[B10] Chen L. P. Wang W. Zhou R. Ng S. C. Li J. Huang M. F. (2014). Characteristics of fecal and mucosa-associated microbiota in chinese patients with inflammatory bowel disease. *Medicine* 93 51–59. 10.1097/MD.0000000000000051 25121355 PMC4602441

[B11] Commission of Chinese Materia Medica (1999). *Chinese Materia Medica* Vol. 8. Shanghai: Shanghai Science and Technology Publishing House, 298.

[B12] Commission of Chinese Pharmacopoeia. (2015). *Pharmacopoeia of the People’s Republic of China.* Beijing: China Medico-Pharmaceutical Science, 253.

[B13] Dziarski R. Park S. Y. Kashyap D. R. Dowd S. E. Gupta D. (2016). *Pglyrp*-regulated gut microflora *Prevotella falsenii, Parabacteroides distasonis* and *Bacteroides eggerthii* enhance and *Alistipes finegoldii* attenuates colitis in mice. *PLOS ONE* 11:e146162. 10.1371/journal.pone.0146162 26727498 PMC4699708

[B14] Flanagan L. Meyer M. Fay J. Curry S. Bacon O. Duessmann H. (2016). Low levels of caspase-3 predict favourable response to 5-FU-based chemotherapy in advanced colorectal cancer: caspase-3 inhibition as a therapeutic approach. *Cell Death Dis.* 7:e2087. 10.1038/cddis.2016.7 26844701 PMC4849164

[B15] Han F. F. Lu Z. Q. Liu Y. F. Xia X. Zhang H. W. Wang X. X. (2016). Cathelicidin-BF ameliorates lipopolysaccharide-induced intestinal epithelial barrier disruption in rat. *Life Sci.* 152 199–209. 10.1016/j.lfs.2016.03.041 27018068

[B16] Horie T. Asada C. Kakigawa N. Sekine S. (2012). Neutrophil infiltration related to the methotrexate-induced intestinal barrier dysfunction. *FASEB J.* 26 1–2.

[B17] Huang Y. L. Zhou Z. H. (2006). Effect of water extracts from Amomi Fruvtus and Millettia Reticulata on the improving the intestinal health and function. *Acad. Period. Farm Prod. Process.* 8 95–98.

[B18] Justino P. F. Melo L. F. Nogueria A. F. Costa J. V. Silva L. M. Santos C. M. (2014). Treatment with *Saccharomyces boulardii* reduces the inflammation and dysfunction of the gastrointestinal tract in 5-fluorouracil-induced intestinal mucositis in mice. *Br. J. Nutr.* 111 1611–1621. 10.1017/S0007114513004248 24503021

[B19] Justino P. F. Melo L. F. Nogueria A. F. Morais C. M. Mendes W. O. Franco A. X. (2015). Regulatory role of *Lactobacillus acidophilus* on inflammation and gastric dysmotility in intestinal mucositis induced by 5-fluorouracil in mice. *Cancer Chemother. Pharmacol.* 75 559–567. 10.1007/s00280-014-2663-x 25572363

[B20] Lam W. Bussom S. Guan F. Jiang Z. L. Zhang W. Gullen E. A. (2010). The four-herb Chinese medicine PHY906 reduces chemotherapy-induced gastrointestinal toxicity. *Sci. Transl. Med.* 2 45–59. 10.1126/scitranslmed.3001270 20720216

[B21] Lee E. J. Kim J. L. Kim Y. H. Kang M. K. Gong J. H. Kang Y. H. (2014). Phloretin protmotes osteoclast apoptosis in murine macrophages and inhibits estrogen deficiency-induced osteoporosis in mice. *Phytomedicine* 21 1208–1215. 10.1016/j.phymed.2014.04.002 24932975

[B22] Lin P. Lu J. M. Wang Y. F. Gu W. Yu J. Zhao R. H. (2015). Naturally occurring stilbenoid TSG reverses non-alcoholic fatty liver diseases via gut-liver axis. *PLOS ONE* 10:e0140346. 10.1371/journal.pone.0140346 26474417 PMC4608713

[B23] Magoč T. Salzberg S. L. (2011). FLASH: fast length adjustment of short reads to improve genome assemblies. *Bioinformatics* 27 2957–2963. 10.1093/bioinformatics/btr507 21903629 PMC3198573

[B24] Mansour-Ghanaei F. Taefeh N. Joukar F. Besharati S. Naghipour M. Nassiri R. (2010). Recurrence of *Helicobacter* pylori infection 1 year after successful eradication: a prospective study in Northern Iran. *Med. Sci. Monit.* 16 144–148. 20190685

[B25] Moorthy G. Murali M. R. Devaraj S. N. (2009). Lactobacilli facilitate maintenance of intestinal membrane integrity during *Shigella dysenteriae* 1 infection in rats. *Nutrition* 25 350–358. 10.1016/j.nut.2008.09.004 19036564

[B27] O’Hara A. M. O’Regan P. Fanning A. O’Mahony C. Macsharry J. Lyons A. (2006). Functional modulation of human intestinal epithelial cell responses by *Bifidobacterium infantis* and *Lactobacillus salivarius*. *Immunology* 118 202–215. 10.1111/j.1365-2567.2006.02358.x 16771855 PMC1782284

[B28] Rinkenbaugh A. L. Baldwin A. S. (2016). The NF-Kb pathway and cancer stem cells. *Cells* 5 2–19. 10.3390/cells5020016 27058560 PMC4931665

[B29] Rowan F. Docherty N. G. Murphy M. Murphy B. Calvin Coffey J. O’Connell P. R. (2010). Desulfovibrio bacterial species are increased in ulcerative colitis. *Dis. Colon Rectum* 53 1530–1536. 10.1007/DCR.0b013e3181f1e620 20940602

[B30] Sheth P. Delos Santos N. Seth A. Larusso N. F. Rao R. K. (2007). Lipopiysaccharide dispruts tight junctions in cholangicocyte monolayers by a c-Src-, TLR4-, and LBP-dependent mechanism. *Am. J. Physiol. Gastrointest. Liver Physiol.* 293 308–318. 10.1152/ajpgi.00582.2006 17446308

[B31] Shukla R. Ghoshal U. Dhole T. N. Ghoshal U. C. (2015). Fecal microbiota in patients with irritable bowel syndrome compared with healthy controls using real-time polymerase chain reaction: an evidence of dysbiosis. *Dig. Dis. Sci.* 60 2953–2962. 10.1007/s10620-015-3607-y 25784074

[B32] Song M. K. Park M. Y. Sung M. K. (2013). 5-Fluorouracil-induced changes of intestinal integrity biomarkers in BALB/C mice. *J. Cancer Prev.* 18 322–329. 10.15430/JCP.2013.18.4.322 25337561 PMC4189444

[B33] Sonis S. T. (2002). The biologic role of nuclear factor-κB in disease and its potential involvement in mucosal injury associated with anti-neoplastic therapy. *Crit. Rev. Oral Biol. Med.* 13 380–389. 10.1177/154411130201300502 12393757

[B34] Sonis S. T. (2004). The pathobiology of mucositis. *Nat. Rev. Cancer* 4 277–284. 10.1038/nrc1318 15057287

[B35] Suzuki T. (2013). Regulation of intestinal epithelial permeability by tight junctions. *Cell. Mol. Life Sci.* 70 631–659. 10.1007/s00018-012-1070-x 22782113 PMC11113843

[B36] Turner J. R. (2009). Intestinal mucosal barrier function in health and disease. *Nat. Rev. Immunol.* 9 799–809. 10.1038/nri2653 19855405

[B37] van Vliet M. J. Harmsen H. J. de Bont E. S. Tissing W. J. (2010). The role of intestinal microbiota in the development and severity of chemotherapy-induced mucositis. *PLOS Pathog.* 6:e1000879. 10.1371/journal.ppat.1000879 20523891 PMC2877735

[B38] Wada M. Nagata S. Saito M. Shimizu T. Yamashiro Y. Matsuki T. (2010). Effects of the enteral administration of *Bifidobacterium breve* on patients undergoing chemotherapy for pediatric malignancies. *Support. Care Cancer* 18 751–759. 10.1007/s00520-009-0711-6 19685085

[B39] Wang J. Jia L. Q. Tan H. Y. Pan L. Yu L. L. Deng B. (2015). Effect of Shengjiang Xiexin Decoction on the repair of damaged rat intestinal mucosa after irinotecan chemotherapy. *Chin. J. Integr. Tradit. West. Med.* 35 1236–1243. 26677677

[B40] Wang J. Yang Z. R. Dong W. G. Zhang J. X. Guo X. F. Song J. (2013). Cooperative inhibitory effect of sinomenine combined with 5-fluorouracil on esophageal carcinoma. *World J Gastroenterol.* 19 8292–8300. 10.3748/wjg.v19.i45.8292 24363520 PMC3857452

[B41] Wang L. Fu T. Lai Y. B. Wang Y. Q. Chen H. L. Wang Y. (2014). Protective effect of curcumin on intestinal mucosal of rat after chemotherapy. *Chin. Arch. Tradit. Chin. Med.* 32 2478–2480.

[B42] Yan Y. Jin M. L. Zhou L. Liu H. F. Chen C. M. Xin Y. (2013). Reulatory effect of herbal medicine Fructus Amomi on antibiotic-induced intestinal flora imbalance in mice. *Chin. J. Microecol.* 25 1040–1043.

[B43] Zhan Y. S. Tan S. W. Mao W. Jiang J. Liu H. L. Wu B. (2014). Chemotherapy mediates intestinal injury via p53/p53 upregulated modulator of apoptosis (PUMA) signaling pathway. *J. Dig. Dis.* 15 425–434. 10.1111/1751-2980.12157 24814616

